# Golgi Phosphoprotein 3 Promotes Colon Cancer Cell Metastasis Through STAT3 and Integrin α3 Pathways

**DOI:** 10.3389/fmolb.2022.808152

**Published:** 2022-03-17

**Authors:** Anpei Huang, Ruizhi Wang, Ji Cui, Ying Gao, Zheng Yin, Lianzhou Chen, Meifang He, Wen Li

**Affiliations:** ^1^ Laboratory of General Surgery, The First Affiliated Hospital, Sun Yat-sen University, Guangzhou, China; ^2^ Department of Laboratory Medicine, The First Affiliated Hospital, Sun Yat-sen University, Guangzhou, China; ^3^ Department of Gastrointestinal Surgery, The First Affiliated Hospital, Sun Yat-sen University, Guangzhou, China

**Keywords:** GOLPH3, stat3, integrin α3, cell invasion and migration, colorectal cancer

## Abstract

**Background:** Golgi phosphoprotein 3 (GOLPH3) overexpression was recently reported to be associated with a poor clinical outcome in patients with colorectal cancer (CRC). However, the underlying molecular mechanism through which GOLPH3 promotes CRC metastasis remains poorly understood.

**Methods:** *In vitro* genetic ablation of GOLPH3 was performed using siRNA transfection, and a stably overexpressed GOLPH3 colon cancer cell line was constructed using the lentivirus system. Cell invasion and migration assays were conducted with or without Matrigel. Immunoblotting, qRT-PCR, immunofluorescence and immunohistochemistry were utilized to study the expression level of GOLPH3, ZEB1, integrin α3 and phosphorylation level of STAT3, AKT/mTOR and Raf/MEK/ERK pathways. Co-immunoprecipitation was used to investigate the interaction between GOLPH3 and p-STAT3 (Tyr705) or total STAT3.

**Results:** Overexpression of GOLPH3 was found in CRC tissues and colon cancer cell lines. Knockdown of GOLPH3 using siRNAs significantly suppressed the invasion and migration of HCT116 and HCT8 cells. In contrast, the overexpression of GOLPH3 promoted the migratory and invasive ability of colon cancer cells. The phosphorylation level of STAT3 as well as the protein and mRNA levels of ZEB1 and integrin α3, were significantly decreased after GOLPH3 knockdown. Moreover, Integrin α3 expression was correlated with GOLPH3 expression in CRC tissues. Co-immunoprecipitation assay revealed that GOLPH3 interacted with pSTAT3 (Tyr705) and total STAT3. Our further experiments suggested that GOLPH3 facilitated IL-6 induced STAT3 activation and subsequently induced transcription of integrin α3 and ZEB1, which promoted the metastasis and progression of CRC.

**Conclusion:** Our current work demonstrates that GOLPH3 facilitates STAT3 activation and regulates the expression of EMT transcription factor ZEB1 and Integrin α3 in colon cancer cells. These findings indicate that GOLPH3 plays a critical role in CRC metastasis and might be a new therapeutic target for CRC treatment.

## Introduction

Colorectal cancer (CRC) is one of the most common digestive malignant tumors that leads to high mortality worldwide, and metastasis is the primary cause of cancer-related death (Dekker et al., 2019; Sung et al., 2021). Therefore, there is an urgent need to unravel the mechanism of tumor metastasis and identify new therapeutic targets for CRC.

Chronic inflammation plays a crucial role in CRC progression (Wang and Sun, 2014). As an inflammatory cytokine from the cancer microenvironment, Interleukin-6 (IL-6) was reported to promote tumor progression via activating multiple signaling pathways, including JAK/STAT3, AKT/mTOR and Raf/MEK/ERK pathways (Kishimoto, 2005). The continuous activation of STAT3 was shown to be a strong promoter of tumorigenesis by stimulating proliferation, survival and metastasis of cancer cells (Bromberg et al., 1999; Yu and Jove, 2004; Yu et al., 2009). STAT3 is aberrantly hyperactive in most malignant cancers, including CRC, and is generally associated with a poor clinical prognosis, suggesting that STAT3 is an important molecular target for CRC therapy (Wang and Sun, 2014; Johnson et al., 2018).

Golgi phosphoprotein 3 (GOLPH3), also known as GPP34, GMx33, MIDAS, or yeast Vps74p, is a highly conserved cytosolic trans-Golgi associated protein from yeast to human (Wu et al., 2000; Bell et al., 2001). Recent studies indicated that high expression of GOLPH3 frequently promotes tumorigenicity and correlates with poor prognosis in different solid tumors, including CRC (Zeng et al., 2012; Zhou et al., 2012; Hu et al., 2013; Hu et al., 2014; Ma et al., 2014; Wang et al., 2014; Zhang L.-J. et al., 2014; Zhang Y. et al., 2014; Guo et al., 2015; Zhou et al., 2016; Zhu et al., 2016). GOLPH3 is a significant contributor to the regulation of tumor cell proliferation, migration and chemoresistance (Sechi et al., 2020). The tumorigenic activity of GOLPH3 is primarily associated with its ability to activate several signaling pathways, such as AKT/mTOR pathway, Wnt/β-catenin pathway and NF-κB pathway (Scott et al., 2009; Dai et al., 2015; He et al., 2019). Qiu et al. reported that overexpression of GOLPH3 activated AKT, which subsequently activated GSK-3β to promote proliferation in colon cancer cells (Zhang et al., 2015). However, the role of GOLPH3 in CRC metastasis remains largely undefined.

IL-6/STAT3 pathway has a pivotal role in metastasis of CRC (Wang and Sun, 2014). Therefore, we focus on the IL-6 mediated signaling pathway to investigate the mechanism of GOLPH3 act in CRC cells invasion and metastasis. Herein, we found that knockdown of GOLPH3 significantly reduced migration and invasion of colon cancer cells, whereas overexpression of GOLPH3 displayed elevated migration and invasion abilities compared to the control groups. We further found that GOLPH3 facilitated IL-6 mediated STAT3 pathway activation by binding to p-STAT3 (Tyr705) and total STAT3. Moreover, GOLPH3 silencing induced the down-regulation of EMT transcription factor ZEB1 and integrin α3 expression. In light of our findings, we provide new evidence that GOLPH3 promotes CRC metastasis via facilitating STAT3 activation and regulating ZEB1 and integrin α3 expression, supporting the use of GOLPH3 as a potential therapeutic target for CRC in the future.

## Materials and Methods

### Patients and Clinical Samples

Surgically resected CRC tissues and adjacent non-cancerous normal tissues were obtained from patients with CRC who had undergone surgical resection in the Gastrointestinal Surgery Department of the First Affiliated Hospital of Sun Yat-sen University (Guangzhou, China). The study was performed with approval from the Clinical Research Ethics Committee of the First Affiliated Hospital at Sun Yat-sen University, and informed consent was obtained from the patients prior to inclusion in this study.

### Cell Culture and Transfection of siRNAs or Lentivirus

Human CRC cell lines HCT116 and HCT8 were purchased from ATCC. HCT116 cells were cultured using McCoy.s 5A Medium (Gibco) with 10% Fetal Bovine Serum (FBS, PΛN), 1% penicillin and streptomycin (Thermo Fisher), while HCT8 cells were cultured in RPMI-1640 Medium (Gibco). All cells were cultured at 37°C with 5% carbon dioxide (CO_2_). The small interfering RNAs (siRNAs) targeting GOLPH3 were purchased from Thermo Fisher Scientific. STAT3, Integrin α3 and control siRNAs (siN0000001-1–5) were purchased from Ribobio (Guangzhou, China). The siRNA sequences for GOLPH3 were 5′-GGA​CCG​CGA​GGG​TTA​CAC​ATC​ATT​T-3’ (siRNA-1) and 5′-GCA​TTG​AGA​GGA​AGG​TTA​CAA​CTA​G-3’ (siRNA-2). The siRNA sequences for STAT3 were 5′-GGC​GTC​CAG​TTC​ACT​ACT​A-3’ (siRNA-1) and 5′-CAT​CGA​GCA​GCT​GAC​TAC​A-3’ (siRNA-2). The siRNA sequences for Integrin α3 were 5′-GGA​CTT​ATC​TGA​GTA​TAG​T-3’ (siRNA-1) and 5′-GAC​CTT​ATC​AAC​CCT​CTC​A-3’ (siRNA-2). Cells were transfected with siRNAs using Lipofectamine RNAiMAX (Thermo Fisher) according to the manufacturer’s instructions. Lentiviral vectors for GOLPH3 overexpression and vector control were purchased from Genecreate (Wuhan, China). Cells were infected with lentivirus containing GOLPH3 or an empty control vector. After 48 h, cells were screened with 1 μg/ml puromycin for 2 weeks. In some experiments, cells were treated with 20 or 50 ng/ml Interleukin 6 (IL-6, Sino Biological) for the indicated time after transfection for 48 h.

### Western Blotting

Cells were lysed with Cell Lysis Buffer (CST) containing protease inhibitor cocktail (Beyotime) and phosphatase inhibitor cocktail (Beyotime). The protein was separated by 10% SDS-PAGE and transferred to PVDF membranes (Roche). The membranes were then blocked with 5% BSA in 1× TBST for 1 h. Subsequently, the membranes were incubated with anti-GOLPH3 (Abcam, ab98023), anti-integrin α3 (ITGA3, Santa Cruz, sc-374242), anti-integrin β1 (ITGB1, Santa Cruz, sc-374429), anti-ZEB1 (CST, 3396), anti-phospho-STAT3 (Tyr705) (CST, 9145), anti-STAT3 (CST, 12640), anti-GAPDH (CST, 5174) overnight at 4°C. GAPDH was used as an internal control. After washing with 1× TBST for three times, the membranes were incubated with goat anti-rabbit IgG HRP (CST) or goat anti-mouse IgG HRP (CST) for 1 h at room temperature and washed with 1× TBST. The immunoreaction signals were detected using the ECL chemiluminescence system (Tanon).

### RNA Extraction, cDNA Synthesis and Real-Time PCR

Total RNA from cells was extracted using Trizol reagent (Thermo Fisher) and reverse-transcribed into cDNA using the PrimeScript RT reagent kit (Takara) according to the manufacturer’s protocol. Quantitative real-time PCR (qRT-PCR) reaction was performed with the Roche 480 system (Roche) using the LightCycler 480 SYBR Green I Master Mix (Roche). The relative mRNA expression levels of target genes were calculated using the 2^−ΔΔCt^ method. GAPDH was used as an endogenous control. The following primer sequences were used: GOLPH3, sense (5′-CTC​CAG​AAA​CGG​TCC​AGA-3′), antisense (5′-CCA​CCA​GGT​TTT​TAG​CTA​ATC​G-3′); ITGA3 sense (5′- TGT​GGC​TTG​GAG​TGA​CTG​TG-3′), antisense (5′- TCA​TTG​CCT​CGC​ACG​TAG​C-3′); ITGB1 sense (5′- CCT​ACT​TCT​GCA​CGA​TGT​GAT​G-3′), antisense (5′- CCT​TTG​CTA​CGG​TTG​GTT​ACA​TT-3′); ZEB1 sense (5′- TTA​CAC​CTT​TGC​ATA​CAG​AAC​CC-3′), antisense (5′- TTT​ACG​ATT​ACA​CCC​AGA​CTG​C-3′); STAT3 sense (5′- CAG​CAG​CTT​GAC​ACA​CGG​TA-3′), antisense (5′- AAA​CAC​CAA​AGT​GGC​ATG​TGA-3′); GAPDH sense (5′- CTG​ACT​TCA​ACA​GCG​ACA​CC-3′), antisense (5′- TGC​TGT​AGC​CAA​ATT​CGT​TG-3′).

### Immunohistochemistry

The colorectal cancer tissue sections were deparaffinized, rehydrated, and 3% H_2_0_2_ was used to block endogenous peroxidase activity. Antigen retrieval was performed by microwave treatment in EDTA (pH 9.0). After blocking with 10% goat serum, the sections were incubated with anti-GOLPH3 (Abcam, ab98023), anti-ITGA3 (Proteintech, 66070-1-ig) overnight at 4°C. Subsequently, the secondary antibodies conjugated with horseradish peroxidase (DAKO) were added for 30 min at 37°C. The sections were detected with 3,3′-diaminobenzidine and counterstained with hematoxylin.

### Immunofluorescence

Cells were grown on glass coverslips in 24-well plates and fixed in 4% paraformaldehyde for 20 min at room temperature. After washing with PBS, the cells were treated with 0.5% Triton-X 100 for 30 min at room temperature to permeabilize cell membranes. Then, the cells were blocked in 3% goat serum with 3% BSA in PBS for 1 h and incubated with anti-ATP1A1 (Proteintect, 14418-1-AP), anti-ITGA3 (Santa Cruz, sc-13545) overnight at 4°C. Subsequently, the cells were incubated with fluorescent-conjugated secondary antibodies against mouse (Alexa Fluor 555, Thermo) and rabbit (Alexa Fluor 488, Thermo) for 1 h at 37°C. ATP1A1 was used as the plasma membrane marker. DAPI (Beyotime) was used to stain and evaluate nuclear morphology.

### Co-Immunoprecipitation

The Flag-tagged GOLPH3 pcDNA3.1 vector was purchased from Genecreate (Wuhan, China). The Flag-tagged construct was transiently transfected into HCT116 cells. After transfection for 48 h, the cells were harvested and lysed using Cell Lysis Buffer (CST) containing protease inhibitor cocktail (Beyotime) and phosphatase inhibitor cocktail (Beyotime). The proteins in the cell lysates were immunoprecipitated using anti-Flag magnet beads (Sigma-Aldrich) at 4°C for 4 h on a rocking plate. The cell lysate and IP-elution were subjected to 10% SDS-PAGE for western blotting analysis.

### Migration and Invasion Assays

Cell migration and invasion assays were performed using a 24 well cell culture insert (8.0 μm pore size, Corning) with Matrigel (invasion) or without Matrigel (migration) according to the manufacturer’s instruction. For migration assays, 2×10^5^ HCT116 cells or 10^5^ HCT8 cells suspended in serum-free medium were seeded into the culture insert. For invasion assays, we used 3×10^5^ HCT116 and 1.5×10^5^ HCT8 cells. The culture inserts were placed into a 24 well plate with a culture medium containing 20% FBS. After incubation for the indicated time (36 h for HCT116 and 20 h for HCT8), the non-migrated cells on the upper surface of the PET membrane were scraped. The migrated cells were fixed with 4% paraformaldehyde and stained with crystal violet. Cell migration photographs were taken, and cells were counted using five random representative fields (×200 magnification; Olympus BX63).

### Statistical Analysis

Data were presented as means ± SEM. Each experiment was repeated at least three times independently. Statistical analysis between two samples was performed using the Student’s t-test. P-values less than 0.05 were considered statistically significant (*p < 0.05, **p < 0.01, ***p < 0.001). All statistical analysis was performed using SPSS 22.0 software (United States).

## Results

### GOLPH3 is Highly Expressed in CRC Tissues and Colon Cancer Cell Lines

**FIGURE 1:**
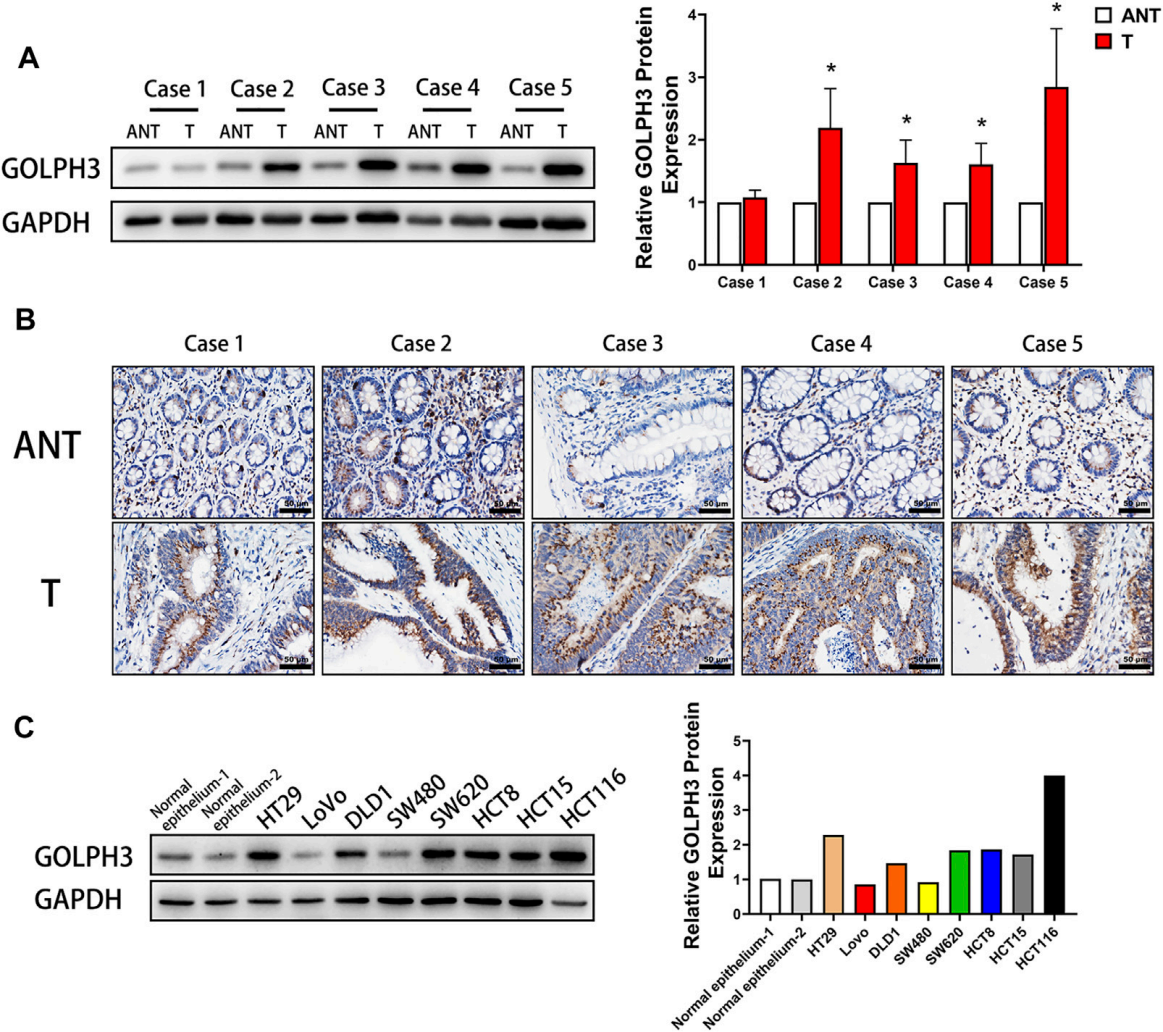
High expression of GOLPH3 in CRC and colon cancer cell lines. **(A)** GOLPH3 protein levels were analyzed in 5 cases of CRC tissues (T) and matched adjacent non-cancerous tissues (ANT) by Western blotting. The bands of GOLPH3 protein were quantified by densitometry and normalized to GAPDH protein. **(B)** Immunohistochemical staining for GOLPH3 in 5 cases of CRC tissues and matched adjacent non-cancerous tissues. **(C)** The expression of GOLPH3 in different colon cancer cell lines were measured using Western blotting. The bands of GOLPH3 protein were quantified by densitometry and normalized to GAPDH protein. For **(A**,**C)**, results were reproduced in three independent experiments, and representative immunoblots are shown. Scale bar, 50 µm *p < 0.05, ***p < 0.001.

To investigate the role of GOLPH3 in CRC progression, we first examined the expression of GOLPH3 in five cases of CRC tissues and matched adjacent non-cancerous tissues by immunoblotting. The results showed that the protein level of GOLPH3 was significantly higher in the four CRC tissues compared to matched adjacent non-cancerous tissues (Figure 1A). Next, immunochemical staining revealed that GOLPH3 was strongly expressed in CRC tissues and was located in the cytoplasm close to the nucleus region. While little or no staining of GOLPH3 was showed in the adjacent non-cancerous tissues (Figure 1B). In six of the eight colon cancer cell lines, the levels of GOLPH3 expression were significantly higher than in normal colon epithelium tissue (Figure 1C). Together, these results demonstrate that GOLPH3 is highly expressed in CRC tissues and colon cancer cell lines.

### GOLPH3 Promotes the Migration and Invasion of Colon Cancer Cells

**FIGURE 2:**
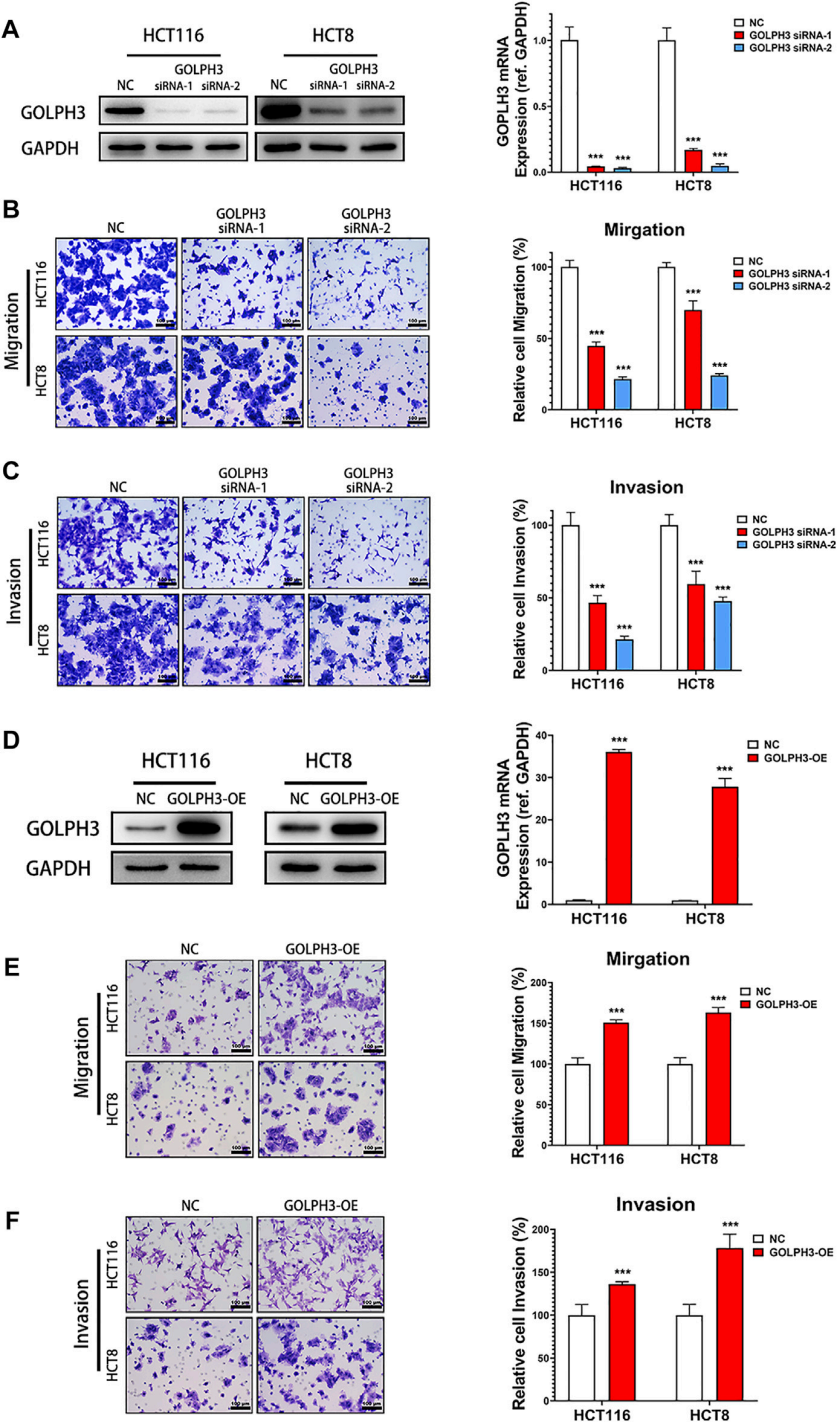
GOLPH3 Knockdown inhibits metastatic phenotypes of colon cancer cells. **(A)** The knockdown efficacy of GOLPH3 siRNAs in HCT116 and HCT8 cells. The GOLPH3 proteins and mRNA levels of control and siRNA transfected cells were measured by western blotting and qRT-PCR, respectively. **(B,C)** The effect of GOLPH3 knockdown in HCT116 and HCT8 cells migration or invasion were examined by transwell assay. The number of migratory or invasive cells were counted and normalized to the NC group in five randomly selected fields. Representative micrographs are also shown (×200 magnification). **(D)** The overexpression efficacy of GOLPH3 in HCT116 and HCT8 cells were measured by western blotting and qRT-PCR, respectively. **(E,F)** The effect of GOLPH3 overexpression in HCT116 and HCT8 cells migration or invasion were detected by transwell assay. The number of migratory or invasive cells were counted and normalized to the NC group in five randomly selected fields. Representative micrographs are also shown (×200 magnification). For **(A**,**D)**, results were reproduced in three independent experiments, and representative immunoblots are shown. Scale bar, 100 µm ***p < 0.001.

To study the function of GOLPH3 in colon cancer cells migration and invasion, we used two independent siRNAs targeting GOLPH3 to knock down its expression in HCT116 and HCT8 cells lines (Figure 2A). Transwell assay showed that knockdown of GOLPH3 significantly suppressed the migratory and invasive abilities of HCT116 and HCT8 cells (Figures 2B,C). To further substantiate the role of GOLPH3 in colon cancer metastasis, we used a lentivirus vector to construct stably overexpressed GOLPH3 in HCT116 and HCT8 cell lines (Figure 2D). As expected, the transwell assay showed that overexpression of GOLPH3 led to significantly enhanced migratory and invasive abilities of HCT116 and HCT8 cells (Figures 2E,F). Taken together, these results confirmed that knockdown of GOLPH3 significantly decreased the migration ability and invasiveness of colon cancer cells, whereas overexpression of GOLPH3 demonstrated the opposite effects.

### Silencing of GOLPH3 Inhibits the Phosphorylation of STAT3 and Expression of ZEB1 and Integrin α3 in Colon Cancer Cells

**FIGURE 3:**
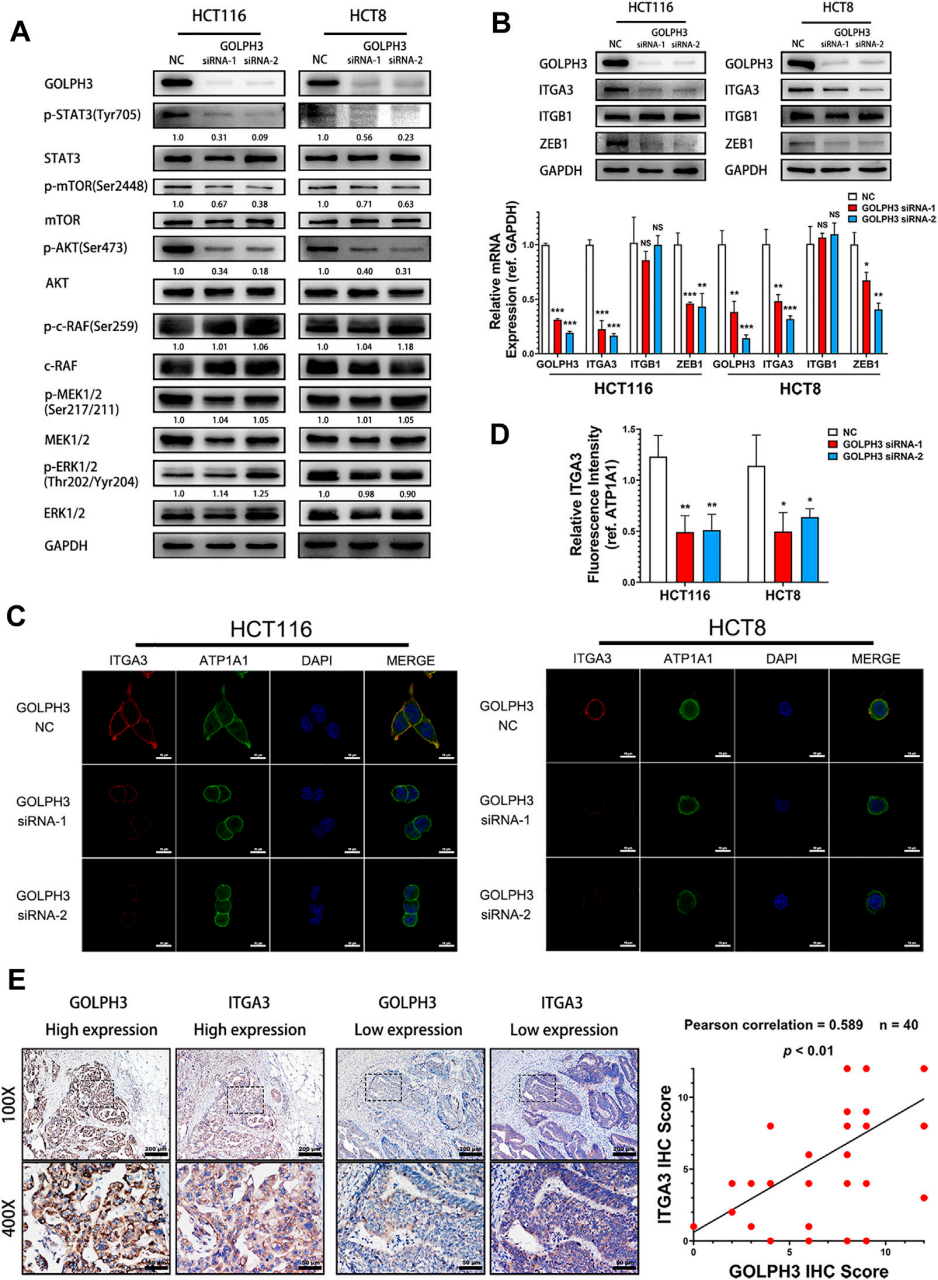
GOLPH3 knockdown suppresses STAT3 phosphorylation and the expression of ZEB1 and integrin α3. **(A)** The phosphorylation level of STAT3 (Tyr705), AKT (Ser473), mTOR (Ser2448), c-Raf(Ser259), MEK1/2(Ser217/211) and ERK1/2(Thr202/Yyr204) of control and GOLPH3 siRNA transfected HCT116 and HCT8 cells were assessed using western blotting. **(B)** Expression of integrin α3, integrin β1 and ZEB1 of control and GOLPH3 siRNA transfected cells were assessed using western blotting and qRT-PCR. **(C,D)** Immunofluorescence staining showed the expression of integrin α3 and ATP1A1 in control and GOLPH3 siRNA transfected cells. ATP1A1 was used as the plasma membrane marker. Scale bar, 100 µm. **(E)** Representative Immunohistochemical images of GOLPH3 and integrin α3 in 40 cases of CRC tissues. The areas in the rectangles in the top columns (100×, Scale bar, 200 µm) were shown at higher magnification (×50, Scale bar, 50 µm) in the bottom columns. Immunohistochemical scores of GOLPH3 and integrin α3 exhibited a positive correlation. For **(A,B)**, results were reproduced in three independent experiments, and representative immunoblots are shown. *p < 0.05, **p < 0.01, ***p < 0.001.

To determine whether GOLPH3 is linked to the IL-6 mediated signaling pathway in CRC metastasis, we first examined the activation of STAT3, AKT/mTOR and Raf/MEK/ERK pathways in GOLPH3 silenced cells. As shown in Figure 3A, knockdown of GOLPH3 resulted in suppression of the phosphorylation of STAT3 (Tyr705), AKT (Ser473) and mTOR (Ser2448), whereas the depletion of GOLPH3 did not affect the phosphorylation of c-Raf(Ser259), MEK1/2(Ser217/211) and ERK1/2(Thr202/Yyr204).

It has been reported that phosphorylation of STAT3 (Tyr705) contributes to ZEB1 up-regulation, which in turn subsequently induces expression of integrin α3 and integrin β1 in pancreatic cancer cells (Liu et al., 2020; Liu et al., 2021). Moreover, phosphorylated AKT could also induce the transcription of ZEB1 via activating β-catenin in hepatocellular cancer (Li et al., 2020). At the same time, integrin α3β1 plays an important role in tumor invasiveness (Kreidberg, 2000; Subbaram and Dipersio, 2011; Ramovs et al., 2017). Therefore, we hypothesized that GOLPH3 might regulate ZEB1 and integrin α3β1 to promote metastasis in CRC patients. As shown in Figure 3B, the protein and mRNA expression levels of integrin α3 and ZEB1 were drastically reduced in GOLPH3 silenced cells. However, the knockdown of GOLPH3 did not affect the expression of integrin β1. Furthermore, immunofluorescence staining showed that the expression of integrin α3 in the cytomembrane was significantly reduced after the silencing of GOLPH3 (Figures 3C,D). In addition, IHC staining analysis of CRC tissues confirmed that patients with higher GOLPH3 expression displayed higher integrin α3 expression compared with patients with lower GOLPH3 expression. Moreover, the GOLPH3 expression level was positively correlated with integrin α3 in CRC tissues (Figure 3E). Taken together, we show that GOLPH3 could promotes the phosphorylation of STAT3 and up-regulate the expression of ZEB1 and integrin α3.

### GOLPH3 Interacts With p-STAT3 to Enhance the IL6-Induced STAT3 Activation in Colon Cancer Cells

**FIGURE 4:**
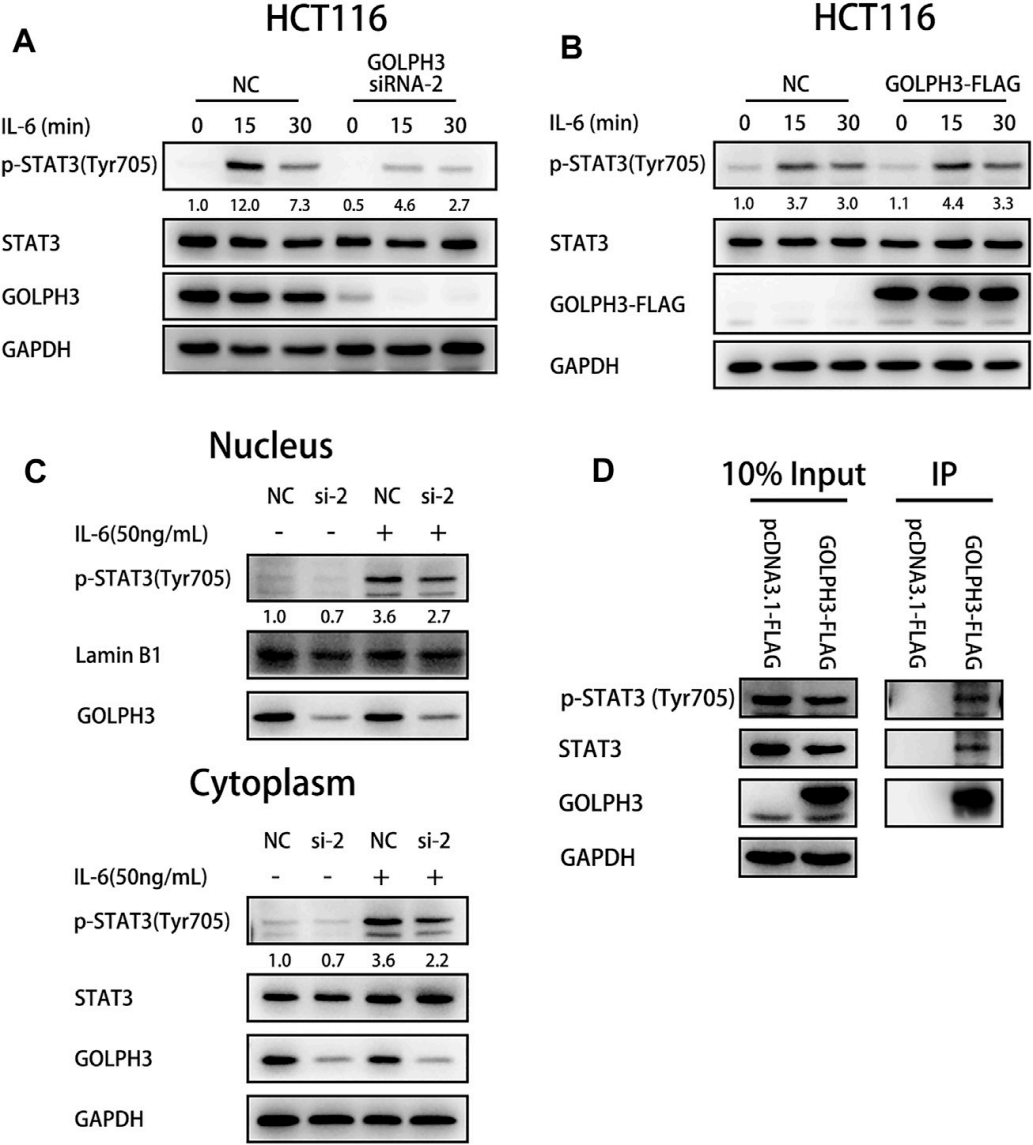
GOLPH3 binds with p-STAT3 to enhance the IL-6 mediated STAT3 activation in HCT116 cells. **(A)** Loss of GOLPH3 suppressed the Tyr705 phosphorylation of STAT3 in HCT116 cells after IL-6 (20 ng/ml) treatment for the indicated times. **(B)** Overexpression of GOLPH3 enhanced the phosphorylation of STAT3 in HCT116 cells after IL-6 (20 ng/ml) treatment for the indicated times. **(C)** HCT116 cells were serum-starved overnight and treated with IL-6 (50 ng/ml) for 30 min. The cells were subjected to nuclear and cytoplasmic fractionation and proteins were analyzed by western blotting. **(D)** The co-immunoprecipitation result showed the interaction between GOLPH3 with p-STAT3 and total STAT3 in HCT116 cells. Results were reproduced in three independent experiments, and representative immunoblots are shown.

To study the function of GOLPH3 in IL-6 mediated STAT3 activation, we treated control and GOLPH3 siRNA-2 knockdown HCT116 cells with IL-6 for 15 or 30 min and examined the phosphorylation of STAT3 at Tyr705. We found that GOLPH3 knockdown significantly attenuated STAT3 phosphorylation at Tyr705 induced by IL-6 (Figure 4A). Furthermore, GOLPH3 overexpression could enhance IL-6 mediated phosphorylation of STAT3 at Tyr705 (Figure 4B). We further examined the cellular distribution of p-STAT3 (Tyr705) in GOLPH3 silenced HCT116 cells after being treated with IL-6 for 30 min. As presented in Figure 4C, the depletion of GOLPH3 could decrease the IL-6 induced p-STAT3 (Tyr705) level in the nucleus and cytoplasm. To address how GOLPH3 regulates the STAT3 pathway, we examined the interaction between GOLPH3 and STAT3 in HCT116 cells. Co-immunoprecipitation assay results indicated that endogenous p-STAT3 (Tyr705) and total STAT3 interacted with Flag-tagged GOLPH3 in HCT116 cell (Figure 4D). These data suggest that GOLPH3 interacts with p-STAT3 and facilitates IL6-induced STAT3 activation in colon cancer cells.

### Silencing of STAT3 Inhibits the Expression of ZEB1 and Integrin α3 in Colon Cancer Cells

**FIGURE 5:**
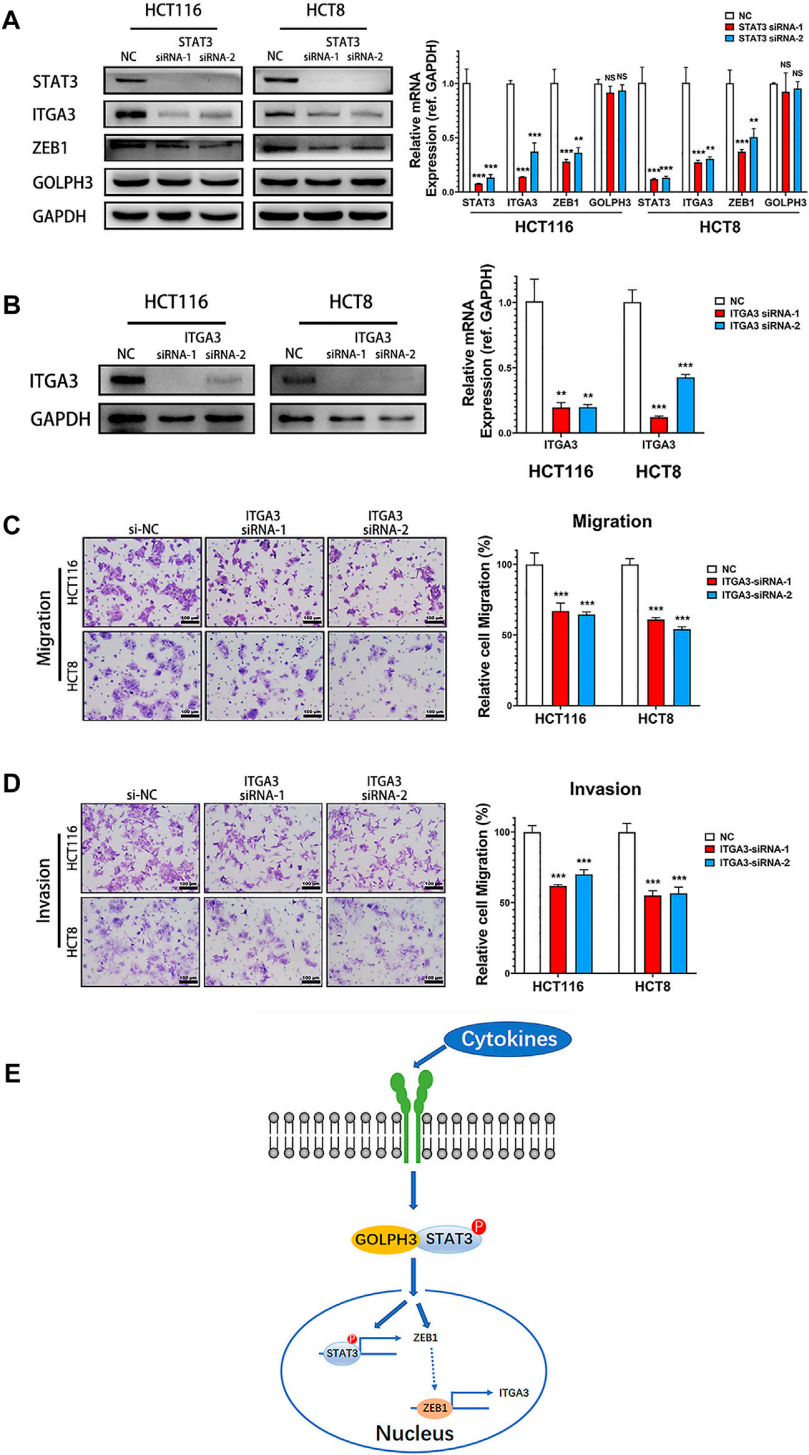
Silencing of STAT3 down-regulated the expression of integrin α3 and knockdown of integrin α3 inhibits metastatic phenotypes of colon cancer cells. **(A)** Expression of ZEB1, integrin α3, and GOLPH3 of control and STAT3 siRNA transfected cells were assessed using western blotting and qRT-PCR. **(B)** Integrin α3 expression in control and integrin α3 siRNA transfected cells were assessed using western blotting and qRT-PCR. **(C,D)** The effect of integrin α3 knockdown in HCT116 and HCT8 cells migration or invasion were examined by transwell assay. The number of migratory or invasive cells were counted and normalized to the NC group in five randomly selected fields. Representative micrographs are also shown (×200 magnification). **(E)** Working model. GOLPH3 interacts with p-STAT3 and regulates ZEB1 and integrin α3 to promote the metastasis of CRC. Scale bar, 100 µm. For **(A,B)**, results were reproduced in three independent experiments, and representative immunoblots are shown. **p < 0.01, ***p < 0.001.

To determine whether the ZEB1 and integrin α3 is regulated by STAT3 signaling in colon cancer cells, we used two independent siRNAs to knock down STAT3’s expression and then examined the expression of ZEB1, integrin α3 and GOLPH3 in HCT116 and HCT8 cells. As shown in Figure 5A, the protein and mRNA expression levels of ZEB1 and integrin α3 were significantly reduced in STAT3 silenced cells. However, there was no change in GOLPH3 level when knocking down STAT3 compared with those in the control cells. These results confirmed that inhibition of STAT3 pathways down-regulated the expression of ZEB1 and integrin α3 in colon cancer cells.

### Knockdown of Integrin α3 Inhibits Migration and Invasion of Colon Cancer Cells

We next detected the effect of integrin α3 on migration and invasion of colon cancer cells. Knockdown of integrin α3 using two independent siRNAs markedly decreased the protein and mRNA level of integrin α3 in HCT116 and HCT8 cells (Figure 5B). Transwell assay showed that integrin α3 knockdown cells migrated more slowly than control cells (Figure 5C). The knockdown of integrin α3 could also significantly reduce the invasive capability of colon cancer cells through the Matrigel membrane (Figure 5D). Together, these results reveal that integrin α3 can promote the migration and invasion of colon cancer cells, which phenocopied the effects of GOLPH3.

## Discussion

Over half of the CRC patients ultimately die from this disease, mainly because most of them already reached the late stage of CRC at initial diagnosis and lack efficacious therapies (Markowitz and Bertagnolli, 2009). Therefore, it is of utmost importance to uncover the molecular mechanism of CRC metastasis and provide new therapeutic strategies. A growing body of research has demonstrated that overexpression of GOLPH3 contributes to tumorigenesis and tumor metastasis (Sechi et al., 2020). In CRC, previous researches demonstrated that GOLPH3 overexpression was significantly associated with tumor invasion depth, lymph node metastasis, distant metastasis and advanced stages (Zhou et al., 2016; Zhu et al., 2016). Patients with GOLPH3 overexpression showed a poor clinical outcome (Wang et al., 2014; Guo et al., 2015; Zhou et al., 2016; Zhu et al., 2016). The 5-years survival rate for patients of GOLPH3 low expression were about 70%, but the rate for patients of GOLPH3 high expression fell to 50% (Guo et al., 2015; Zhu et al., 2016). Those results showed that overexpression of GOLPH3 plays a crucial role in tumor metastasis and poor survival of CRC patients. However, the molecular mechanisms that GOLPH3 utilizes to promote CRC metastasis are poorly understood.

Elevated levels of IL-6 in serum have been associated with advanced stages, metastasis and poor clinical outcome in CRC patients (Knupfer and Preiss, 2010). Grivennikov et al. revealed that continuous stimulation of IL-6 was required for colitis-associated colon cancer in mice models (Grivennikov et al., 2009). In addition, STAT3 is a critical IL-6 effector that induces colitis-associated colon cancer (Grivennikov et al., 2009). Several studies have revealed that the invasive behavior of CRC cells was closely associated with the IL-6/STAT3 pathway (Rokavec et al., 2014; Wang and Sun, 2014; Wei et al., 2019). Our study found that loss of GOLPH3 attenuated the IL-6 induced phosphorylation of STAT3 in colon cancer cells, while the overexpression of GOLPH3 enhanced STAT3 phosphorylation in the presence of IL-6. Furthermore, the co-immunoprecipitation assay confirmed that GOLPH3 interacted with p-STAT3 (Tyr705) and total STAT3 in colon cancer cells, which indicates that GOLPH3 might act as a transport protein. GOLPH3 is involved in regulating Golgi-to-plasma protein trafficking, endocytic trafficking and contributes to malignant phenotypes via interacting with RAB1B, RAB5, MYO18A and coat protein complex I (COPI) (Dippold et al., 2009; Eckert et al., 2014; Halberg et al., 2016; Zhou et al., 2017). In glioma, GOLPH3 was found to facilitates the interaction of JAK2 and STAT3, leading to activation of the STAT3 pathway (Wu et al., 2018). Previously, we reported that GOLPH3 interacted with STIP1, which then regulated telomerase activity and promoted tumor progression in pancreatic ductal adenocarcinama (Wang et al., 2020). However, whether GOLPH3 regulates Golgi-to-nuclear protein trafficking is still not clear. Our present study indicated that GOLPH3 could binds and facilitates p-STAT3 from the cytoplasm into the nucleus to activate the downstream pathway. Moreover, we showed that loss of GOLPH3 suppressed the activation of STAT3 and AKT-mTOR pathways, suggesting that GOLPH3 might be involved in the crosstalk between the STAT3 pathway and AKT-mTOR pathway.

STAT3 and AKT phosphorylation contributes to the up-regulation of several EMT transcription factors and enables further activation of the EMT process (Zhao et al., 2015; Saitoh et al., 2016; Karimi Roshan et al., 2019; Liu et al., 2020). Phosphorylated STAT3 can directly binds to the promoter region of ZEB1 and induces the transcription of ZEB1 in pancreatic cancer cells (Liu et al., 2020). Moreover, Li et al. revealed that the phosphorylation of AKT promoted nuclear translocation of β-catenin to initiate transcription of ZEB1 (Li et al., 2020). ZEB1 is a key EMT transcription factor that acts as a transcription repressor to suppress E-cadherin’s epithelial factors during the EMT process (Caramel et al., 2018). Our study found that the silencing of GOLPH3 down-regulated the expression of ZEB1, EMT transcription factor Snail, mesenchymal marker N-Cadherin, and up-regulated epithelial factors E-cadherin (data not shown). Those results indicate that GOLPH3 might promotes the metastasis of CRC by regulating the phenotypes of EMT through ZEB1.

ZEB1 can also activate downstream target transcription by interacting with coactivators, such as YAP1 (Liu et al., 2021). Liu et al. demonstrated that ZEB1 could binds to integrin α3β1 promoter regions and forms a complex with YAP1 to activate integrin α3 transcription through the YAP1/TEAD binding sites in pancreatic cancer cells (Liu et al., 2020; Liu et al., 2021). Integrin α3β1 has been previously linked to tumor progression and metastasis in CRC (Qin et al., 2020; Tian et al., 2020). Herein, the knockdown of GOLPH3 expression markedly down-regulated the expression of integrin α3, while there was no change in the expression of integrin β1. The silencing of integrin α3 led to a decrease in colon cancer cells’ invasion and migration abilities. In addition, we found that GOLPH3 expression correlated positively with integrin α3 expression in CRC tissues. These findings suggest that GOLPH3 may promote the invasion and migration of colon cancer cells via regulating integrin α3 expression. Interestingly, Isaji et al. revealed that GOLPH3 interacted with sialyltransferases to regulated N-glycosylation of integrin β1 and enhanced integrin-mediated cell migration in breast cancer (Isaji et al., 2014). Furthermore, phosphatidylinositol 4-kinase IIα, which is one of the regulators of GOLPH3, regulates N-glycan sialylation of integrin α3β1 and integrin α3 mediated cell migration via binding with integrin α3β1 (Isaji et al., 2019). Integrin α3 and integrin β1 are glycosylated and fully processed heterodimers in the Golgi before being transferred to the cell surface (Rainero and Norman, 2013). In comparison, GOLPH3 is important for localizing several glycosyltransferases in the Golgi and regulates Golgi-to-plasma protein trafficking (Schmitz et al., 2008; Dippold et al., 2009; Eckert et al., 2014; Pereira et al., 2014). Therefore, we surmise that GOLPH3 not only regulates the transcription of Integrin α3 but is also involved in the glycosylation and Golgi-to-plasma trafficking of integrin α3. Further investigation on the relationship between GOLPH3 expression and integrins mediated cell migration is needed to gain more insight into GOLPH3’s role.

In summary, this study provides evidence that GOLPH3 is involved in regulating colon cancer cell invasion and migration through the STAT3 pathway and silencing GOLPH3 down-regulates ZEB1 and Integrin α3 expression to prevent CRC metastasis (Figure 5E). Therefore, our findings suggest that GOLPH3 is an important oncogene that promotes CRC metastasis. Further in-depth research on the functional role of GOLPH3 function is warranted to provide new insight for developing novel therapeutic targets to treat CRC metastasis.

## Data Availability

The raw data supporting the conclusion of this article will be made available by the authors, without undue reservation.

## Abbreviations

ANT, the adjacent non-cancerous tissues; CRC, colorectal cancer; GOLPH3, Golgi Phosphoprotein 3; EMT, epithelial-mesenchymal transition; IL-6, Interleukin-6; STAT3, signal transducer and activator of transcription 3; T, tumor tissues; ZEB1, zinc dependent EMT like transcription factor 1.
